# “Desperate not to make the same mistakes”: Couple adjustment to parenthood in the context of childhood maltreatment

**DOI:** 10.1002/imhj.70106

**Published:** 2026-06-15

**Authors:** Laura N. Martin, Keith D. Renshaw, A. Grace Kelly, Debora G. Goldberg

**Affiliations:** ^1^ George Mason University Fairfax Virginia USA

**Keywords:** childhood maltreatment, coparenting, qualitative research, resilience, romantic partners, transition to parenthood

## Abstract

This study explored how a history of childhood maltreatment shapes the transition to parenthood, with a focus on how partners jointly negotiate this adjustment within the couple relationship. Using Interpretative Phenomenological Analysis (IPA), in‐depth semi‐structured interviews were conducted with 11 heterosexual couples (22 individuals) living in the United States, in which at least one partner reported a history of childhood maltreatment. Interviews were analyzed for emergent themes related to adjustment to parenthood, relational functioning, and dyadic support processes. Four overarching dyadic themes emerged: (1) relational meaning‐making of childhood maltreatment, (2) relational challenges activated by maltreatment history, (3) dyadic support as co‐regulation for maltreatment‐related vulnerability, and (4) couples’ resilient and intentional orientation toward family life. Findings suggest that supportive romantic partnerships may function as a relational context through which parenting‐related self‐doubt is negotiated, emotional safety is fostered, and caregiving approaches distinct from participants’ own childhood experiences are collaboratively constructed. These insights have implications for dyadic interventions during the transition to parenthood that aim to support reflective parenting and relational resilience.

## 1 INTRODUCTION

Becoming parents is one of the most significant transitions many couples will experience, marked by heightened stress, identity shifts, and lifestyle changes. While most couples experience declines in relationship quality during this transition, some report stability or improvement (Belsky & Kelly, 1994; Belsky & Rovine, 1990; Doss et al., 2009). Much research on couples during the transition to parenthood focuses on risk factors for negative adjustment (Kluwer et al., 2010). Traumatic experiences, such as childhood maltreatment, are linked to poor adjustment during the transition to parenthood (Belsky & Isabella, 1985), including increased parenting stress, mental health challenges, and disruptions in parent–child bonding (Racine et al., 2021). This may be due to the anticipation of caregiving responsibilities activating internal working models of attachment and caregiving formed in one's own childhood (Cowan & Cowan, 2000). Research suggests that becoming a parent for the first time represents a developmental period in which past relational experiences are reevaluated and may shape expectations about parenting and coparenting roles (George & Solomon, 2008). As such, primiparous couples may be uniquely likely to reflect upon and renegotiate the influence of childhood maltreatment on their emerging parenting identities.

**Key Findings**

- **Partners supported relational meaning‐making of maltreatment history**
  Romantic partners often played a critical role in helping individuals recognize, validate, and reinterpret childhood maltreatment experiences. This shared meaning‐making deepened emotional understanding within the relationship and informed expectations for parenting.
- **Couples engaged in dyadic support processes that regulated parenting‐related vulnerability**
  Despite challenges, couples described engaging in relational support strategies—including affirmation, collaborative boundary‐setting, and flexible coparenting—that helped manage parenting‐related self‐doubt linked to earlier caregiving experiences.
- **Couples approached parenting with resilience and shared intentionality**
  Rather than repeating negative patterns, many couples described jointly reflecting on how they wished to parent differently from their own caregivers and approaching parenting with intentionality. Supportive partnerships were experienced as reinforcing confidence in adapting to the parenting role together.

Although evidence suggests that a history of childhood maltreatment may have negative impacts on an individual's romantic partner, existing research has not explored these impacts within the context of transition to parenthood (Vaillancourt‐Morel et al., 2024). The negative association of a history of childhood maltreatment with adjustment in mothers during pregnancy and the postpartum period is well‐established, including an increased negative sense of self‐worth, feelings of guilt and shame, experiencing a lack of control within the context of parenting, experiencing stigma, lacking parenting knowledge and skills, and broad mental and physical health problems (see Chamberlain et al., 2019a for review). Despite this robust set of findings on maltreatment's effect on mothers, less research focuses on how maltreatment impacts couples during this time.

**Statement of Relevance**

This study offers nuanced insight into how childhood maltreatment impacts the transition to parenthood within the romantic and coparenting relationships. By centering both members of the couple, this work highlights the role of partners in supporting relational meaning‐making of earlier caregiving experiences and regulating parenting‐related vulnerability during this transition. Findings contribute to infant mental health by illuminating dyadic processes that buffer the impact of early adversity and support the development of the emerging family system during a critical developmental window.

Posttraumatic change refers to shifts in individuals’ beliefs, identities, or relational patterns that emerge following traumatic experiences and may include both positive and negative adaptations. While many of the posttraumatic changes that occur after maltreatment are negative, some report their maltreatment experiences impact them in a more nuanced way. Fava & colleagues (2016) coded interviews with new mothers to understand perceptions of general and parenting‐specific posttraumatic change after childhood maltreatment. Three‐quarters of mothers reported that general posttraumatic changes included a mix of positive and negative changes, with 8% reporting negative changes only (17% reported positive changes only). Moreover, parenting‐specific posttraumatic changes, such as being able to connect with and understand your child more due to past experiences, were even more likely to be perceived as wholly positive. This variability suggests protective factors exist and can inform interventions for parents with childhood maltreatment histories.

Most of the studies examining the transition to parenthood after childhood maltreatment are quantitative and only examine mother's experiences (see Chamberlain et al., 2019a; Christie et al., 2017 for reviews). A predominant finding is that positive relationships with intimate partners and social support serve as protective factors for mothers with maltreatment histories during the perinatal period (Chamberlain et al., 2019a). Similarly, within the handful of qualitative studies (most of which focus on women with a history of sexual abuse), positive trajectories are often associated with the relationships and environments these mothers inhabit rather than being individual processes. For instance, themes such as *creating safety* (building safe relationships and places for their baby), *compassionate care* (feeling valued and cared for), and *feeling connected* (quality of relationships during the transition) rely on support systems (Chamberlain et al., 2019b). Prior research also highlights the complexity of intimate partner relationships in this context, as some survivors report conflict, misunderstanding, or even intimate partner violence during the transition to parenthood (Chamberlain et al., 2019b). Thus, the quality of the romantic relationship may impact adjustment to parenthood in individuals with a history of childhood maltreatment. Recognizing this variability underscores the importance of examining romantic relationships as a critical determinant of parental adjustment and well‐being during the transition to parenthood

Disclosure and communication about maltreatment between partners may play a role in their adjustment during the transition to parenthood. Research suggests that the majority of abuse survivors disclose their experiences to their partners (Rubinsky et al., 2023; Ullman, 2002), with some finding it empowering, engendering more support and sensitivity from others, whereas others experienced feelings of being judged or stigmatized (Palmer, 2004). In one qualitative study of female survivors of sexual abuse, several mothers expressed a reluctance to discuss past maltreatment, with one mother stating that it would make her pregnancy feel “tainted with memories of a dirty past,” (Palmer, 2004, p. 162). Despite this reluctance, avoiding discussion was not tied to fewer thoughts about maltreatment during pregnancy, and many of these women still experienced distress during this time. While these findings suggest that disclosure of maltreatment may impact adjustment to parenthood, no study has examined communication about maltreatment with partners during the transition to parenthood.

Partner responses to disclosure are crucial, as negative reactions may worsen psychological symptoms (Ullman, 2002). In qualitative studies of the transition to parenthood, feeling supported and not judged by one's partner helped women to feel more positive about their pregnancy (Chamberlain et al., 2019b), whereas others experienced partners as judgmental about trauma‐related symptoms during this transition (Berman et al., 2014). Considering the importance of partners’ reactions to disclosure of maltreatment, it is possible that having a partner who had a similar childhood may be beneficial due to increased empathy for the many ways in which the transition to parenthood could be re‐traumatizing. While preliminary quantitative results suggest compounded negative effects when both partners have maltreatment histories (Martin et al., 2025), more research is needed to draw firm conclusions.

Finally, adjustment to parenthood can be influenced by incongruence between expectations and reality for parents, particularly for parents with a maltreatment history whose views of themselves may be distorted (Fitzgerald et al., 2005). These distorted self‐perceptions may be reinforced by their support network. A qualitative study of childhood sexual abuse survivors’ experiences during pregnancy highlighted how new parents may consider their partner's upbringing when assessing parenting ability (Lasiuk, 2007). One participant highlighted how her view of her non‐maltreated partner's upbringing reassured her that he would be a “good dad” (Lasiuk, 2007, p. 184). However, little is known about how non‐maltreated partners view their maltreated partner's suitability for parenting how this may affect both partners’ adjustment to parenthood.

Because of the tendency to assume the sequelae of maltreatment are negative, quantitative studies may put undue emphasis on negative outcomes rather than capturing the nuance of how a maltreatment history impacts new parents. Qualitative methods allow individuals to share a range of attributions and perceptions related to the transition to parenthood and how their maltreatment history impacted that transition. This study employs a qualitative approach to explore how maltreated parents and their partners adjust to parenthood, with a focus on relationship functioning as a potential protective factor. Despite existing qualitative research on perinatal experiences, few studies include both mothers and fathers, center on relationship quality, or examine both partners in couples where one parent was maltreated (Chamberlain et al., 2019b). By studying couples rather than individuals, this research takes a more naturalistic approach, recognizing that parenthood is rarely experienced in isolation.

### 1.1 Study aims and research questions

This study aims to explore how a history of childhood maltreatment shapes couple dynamics and support during the transition to parenthood using a discovery‐oriented approach. The research questions guiding this study are:

1. What is the lived experience of couples where at least one partner has a history of childhood maltreatment during the transition to parenthood?
2. How do these couples support each other's needs related to maltreatment during the transition to parenthood? How does this support influence their adjustment?

## 2 METHOD

### 2.1 Interpretive phenomenological analysis

To explore these research questions, interpretive phenomenological analysis (IPA) was selected as the most suitable approach (Smith & Osborn, 2015; Smith et al., 2009). Drawing from phenomenology, IPA is a qualitative approach that aims to generate contextualized knowledge to provide novel insight into individuals’ lived experiences. IPA acknowledges that interpretation is happening at two levels (double hermeneutics)—the researcher trying to make sense of what the participants are sharing, and the participants themselves trying to make sense out of their own experiences (Smith et al., 2009). Other studies focused on similar topics have used this methodology (e.g., Bécotte et al., 2023; Ngameni et al., 2022).

### 2.2 Participants

Study inclusion criteria included: having given birth to one's first child within the past 12 months, being married or in a committed relationship (dating for more than 18 months or cohabitating), at least one parent having a history of childhood maltreatment (assessed using the Childhood Trauma Questionnaire's (CTQ) established cutoffs for any clinically significant form of maltreatment; Bernstein et al., 1994), and both members of the couple consenting to participate in the study. Further, couples had to be English‐speaking and able to participate in online interviews over Zoom, a secure telecommunication platform.

Eleven mixed‐sex couples were interviewed for the study. All couples were married, had their first child together in the past year, and lived together at the time of the interview. The average age was 33.82 years (*SD *= 7.21) for men and 31.00 years (*SD *= 5.10) for women. Four out of 11 couples had two partners who endorsed experiencing some form of childhood maltreatment, while the other seven couples only had one partner (3 women and 4 men) who endorsed maltreatment. Among the 15 participants who met criteria for clinical levels of childhood maltreatment on the CTQ (CTQ; Bernstein et al., 1994), the average childhood maltreatment score was 40.00 (*SD *= 11.42). Among participants who endorsed maltreatment, most reported at least low levels of emotional abuse (80%; *M* = 10.53, SD = 4.39, range = 5–23) and emotional neglect (73%; *M* = 11.07, SD = 4.89, range = 5–24). Fewer reported physical abuse (47%; *M* = 6.60, SD = 1.80, range = 5–9), sexual abuse (20%), and physical neglect (13%; *M* = 6.33, SD = 2.20, range = 5–14). The average age of the infant at the time of the interview was 7.45 months (*SD *= 2.36), ranging from 4–12 months. Couples lived in 8 different states. More sample characteristics can be found in Table 1.

**TABLE 1:** Participant characteristics (*N* = 22).

Characteristics of participants	*N* (%)
**Child age (*N* = 11)**	
4–6 months	4 (36.4%)
7–9 months	4 (36.4%)
10–12 months	3 (27.3%)
**Participant age (years)**	
20 to 24	1 (4.5%)
25 to 29	9 (40.9%)
30 to 34	4 (18.2%)
35 to 39	6 (27.3%)
40 to 44	1 (4.5%)
45 to 49	1 (4.5%)
**Household income**	
$20,000–$50,000	2 (9.1%)
$50,000–$100,000	4 (18.2%)
$100,000–$150,000	5 (22.7%)
$150,000 or more	11 (50.0%)
**Race**	
White/Caucasian	14 (63.6%)
Asian (Asian Indian, Chinese, etc.)	1 (4.5%)
Hispanic/Latino/Latinx	4 (18.2%)
Multiracial or Biracial	3 (13.6%)
**Education**	
Associate's degree	3 (13.6%)
Bachelor's degree	8 (36.4%)
Master's degree	5 (22.7%)
Doctoral or professional degree	6 (27.3%)
**United states region**	
Northeast	2 (9.1%)
Mid‐Atlantic	10 (45.5%)
South	8 (36.4%)
Midwest	2 (9.1%)

### 2.3 Procedures

All study procedures were reviewed and approved by the Institutional Review Board at the authors’ affiliated institution, and all participants provided informed consent prior to participation. Data was collected through semi‐structured interviews with primiparous couples between 0 and 12 months postpartum. Participants were recruited via purposeful sampling (Patton, 2002), through obstetrics clinics in the mid‐Atlantic region, as well as online via postings in targeted social media and pregnancy/parenting groups. Purposeful sampling focuses on recruitment of participants who are especially knowledgeable about or experienced with the phenomenon of interest (Creswell & Plano Clark, 2011). Postpartum couples were able to report retrospectively on various phases of their adjustment to parenthood and changes in relationship factors across time (e.g., alignment between expectations for parenting during pregnancy and actual parenting experiences). Primiparous couples were prioritized because the influence of childhood maltreatment is likely most salient during the transition to first‐time parenthood (George & Solomon, 2008).

Inclusion criteria were assessed using an online screener that was sent out via email prior to participants enrolling in the study. The screener survey included the CTQ (CTQ; Bernstein et al., 1994) to establish a history of maltreatment and a brief demographic questionnaire to establish language, age, length of relationship, and months postpartum. Both partners completed the screener survey separately. Couples who were deemed eligible to participate based on the screener survey were then contacted by the research team to schedule interviews.

Semi‐structured interviews were conducted with each partner individually, and couples were compensated $50 total ($25 per partner) after both partners completed their interviews. Partners were interviewed separately to encourage open reflection on sensitive experiences and to capture each partner's individual meaning‐making prior to examining dyadic patterns across interviews. Interviews were expected to be 60 min each but varied depending on the length of responses. Data were collected via audio‐recorded interviews conducted over Zoom, and transcripts were generated using transcription software. Transcripts were checked against audio recordings for reliability and then uploaded into the MAXQDA, which was used for data management, coding, and analysis. After interviews and coding were completed, member checking was conducted. Participants were sent a written summary of the themes and subthemes found in the data and given the opportunity to provide feedback to the research team. Participant feedback was incorporated into the analysis below.

IPA analysis does not have a universal rule of thumb for sample sizes; rather, sample size varies based on the ability to purposefully sample participants who have experienced the phenomenon of interest (Pietkiewicz & Smith, 2014). IPA studies have been published with a range of participants, but within clinical psychology, the recommended number is 6 to 8 participants (Turpin et al., 1997). Our recruitment target was 10 couples, as couples were the unit of analysis. Recruitment continued until saturation was reached, or when additional interviews no longer meaningfully extended or challenged existing interpretive insights, while still honoring the idiographic uniqueness of each couple (Roberts, 2014).

### 2.4 Measures

#### 2.4.1 Childhood Trauma Questionnaire

Each parent completed the 28‐item Childhood Trauma Questionnaire (CTQ; Bernstein et al., 1994), a self‐report measure that contains five subscales (emotional abuse, physical abuse, sexual abuse, emotional neglect, and physical neglect), each with 5 items, and a 3‐item Minimization/Denial scale. Respondents rate items on a 5‐point Likert scale from 1 (never true) to 5 (very often true). Participants met inclusion criteria if they reached cutoffs indicating at least “low” levels of maltreatment on at least one of the five subscales (Bernstein et al., 1994).

#### 2.4.2 Interview

The first author conducted semi‐structured Zoom interviews between July 2023 and April 2024. Interviews were conducted virtually to increase feasibility for parents with infants at home and reduce barriers to participation. The average interview length was 64 min (range: 53–85 min). The full interview guide can be found in Appendix A. Interviews intentionally involved minimal probing to allow participants to guide the focus and depth of discussion, preserving the participants' own sense‐making process rather than pushing the research team's a priori interpretations. The interviewer frequently checked with participants as to whether there was any information they viewed as pertinent to their experience that was not discussed, leaving time to explore these areas.

### 2.5 Analytic plan

Interview transcripts were coded by the first author using the method outlined by Finlay (2011), following two key levels of coding. First, each transcript was replayed and read to re‐familiarize the first author with the material. Then, first‐order analysis, or initial/open coding, was completed, focused on annotating each transcript to attach concepts to sections within interviews. Memoing was also conducted to capture ideas derived from the data to identify processes that are occurring. After analysis of each individual transcript, paired partner transcripts were examined together as a dyad to explore convergence, divergence, and co‐constructed meaning around childhood maltreatment and the transition to parenthood.

Next, second‐order analysis occurred, moving beyond pure description toward interpretation. This involved creating categories or higher‐order concepts, which assign meaning and the researcher's interpretation to the lower‐order concepts that emerged in initial coding. Categories were created by looking at both reoccurring themes across couples and themes within couples, particularly mirrored or shared experiences and asymmetries between partners within couples. The first author engaged in the double hermeneutic, attempting to make sense of participants making sense of their own experiences (Smith et al., 2009), by then re‐examining early interpretations in memos made during interviews and in initial transcript reads through the lens of the higher order analysis.

These codes were then discussed with the research team to understand broader patterns occurring and novel experiences each participant described. To reduce biases during the analysis, the first author met regularly with the research team and other authors to share her analytic process and reflect on the themes discovered. The research team, including the first author, consisted of 5 women, 3 who identified as white, 1 who identified as Central Asian, and 1 who identified as mixed race. The first author and 1 other member of the research team have children of their own. Given that some members of the research team were parents themselves, reflexive discussions attended to how assumptions about “healthy” parenting or couple functioning might shape interpretation, particularly when analyzing how well participants have adjusted to parenthood.

The research team then reviewed the coding completed by the first author and reread each transcript to identify any themes or concepts that had been missed. Overall, the two stages of coding aligned with IPA's idiographic nature, allowing each couple's experiences to be identified and analyzed before drawing connections between shared experiences (Miller et al., 2018).

## 3 RESULTS

**FIGURE 1:**
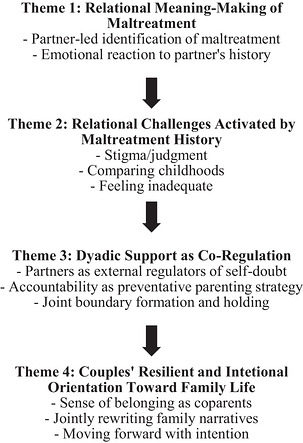
Theme map illustrating dyadic processes through which couples with histories of childhood maltreatment navigate the transition to parenthood.

Analysis revealed four interrelated dyadic processes through which couples navigated the transition to parenthood in the context of childhood maltreatment: (1) making sense of maltreatment within the couple, (2) relational challenges activated by maltreatment history, (3) dyadic support as co‐regulation for maltreatment‐related vulnerability, and (4) couples’ resilient and intentional orientation towards family life. Figure 1 illustrates how couples moved from shared meaning‐making of maltreatment experiences to intentional approaches to parenting. Additional representative quotes can be found in Appendix B.

### 3.1 Theme 1: Relational meaning‐making of childhood maltreatment

Many couples shared that being in a relationship created new contexts in which earlier maltreatment experiences were named, reinterpreted, and processed together. This phenomenon was captured well by one participant:

I think there's like a saying that being married is like having a mirror held up to you. So, [Partner] will point things out, and he'll be like, ‘That's just like your mom.’ (38b)

#### 3.1.1 Partner‐led identification of maltreatment

Across all couples, the maltreated partner disclosed their maltreatment experiences, yet almost all engaged in some level of minimization with their partner. Participants often contrasted their experiences with more extreme forms of abuse or neglect, implicitly positioning their own suffering as less legitimate. Some participants were aware of the downplaying—“I know a lot of kids make excuses for their parents too, so I could be doing that as well” (23b). Participants often explained this minimization as a way of reconciling continued relationships with caregivers or justifying their caregivers’ behaviors through the context of their own difficult childhoods. Finally, some noted that they minimized or withheld their experiences due to fear that their partner would judge their parents or caregivers.

At the couple level, this downplaying often became a site of identification and making sense of maltreatment. About half of the couples shared that one partner had to identify the other partner's experience of maltreatment as such. A common theme was participants not realizing until they disclosed their childhood experiences to their partner that their experiences reflected maltreatment. Partners 36a and 36b both described feeling validated when their partner was witness to their own maltreatment experience: “I think the benefit for me was … validation that some of the things that happened were ridiculous. Or things that shouldn't have happened” (36b). Partner 36b described her husband's validation being more valuable than that of a therapist, since he witnesses the treatment, she receives from her caregivers. Similarly, Partner 36a described his wife being on speaker phone and hearing the way his mother speaks to him as helpful in identifying maltreatment. This suggests that there is a unique lens through which a romantic partner can help provide identification and validation of the maltreatment experience as abnormal.

#### 3.1.2 Emotional reactions to partner's history

Another important aspect was participants’ experience of learning about their partner's maltreatment history. Many partners expressed feelings of anger or sadness about their partner's maltreatment.

It hurts me when he talks about how he was talked to as a child and like thinking about how you can just like belittle someone all the time… it does make me really sad that he didn't have the support when he was younger. (34b)

Two partners shared that learning about their partner's maltreatment made them feel luckier and more appreciative of their positive childhood experiences.

Understanding more of where she came from increased my respect and trust for her, and that this is who she was able to become, despite all those challenges. I think it motivated me to some degree to want to do even better because, hey, I was handed this really great hand, you know, am I going to waste it? (38a)

Although the overwhelming response by partners was supportive and validating, two of seven non‐maltreated participants shared that learning about their partner's maltreatment history led to initial concerns about their partner's ability to parent or their relationship patterns. For instance, Participant 23a explained that he worried that there was something in him that reminded his wife of her father, who had maltreated her, and that their relationship would recreate some of those negative patterns.

Almost all couples still had some level of interaction or contact with the maltreating caregivers, adding a level of complexity to the partner's response to learning about the maltreatment. Partners had to both navigate validating their spouse's negative experience while also supporting their partner's ongoing relationships with those same caregivers (see *Joint Boundary Formation and Holding*). Participant 38a explained feeling “angry and resentful against” his wife's mother and noted, “I think [my wife's] experience of [my] anger was almost exclusively hurtful… I think it took some time for her to understand that when I was voicing frustration or anger at her mom, I wasn't attacking her, but because of her experience, that did not come naturally.”

### 3.2 Theme 2: Relational challenges activated by maltreatment history

Although challenges related to maltreatment history often emerged prior to pregnancy, participants described these issues as becoming more salient in the context of discussions about having children. Across several couples, difficulties with emotion regulation or communication that participants attributed to their upbringing became increasingly important to address when considering parenthood.

I think there was a time where I was like, if he doesn't get it together and get this anger under control, I'm going to leave because I don't want my children to grow up with someone that's angry and who will treat other people this way. (34a)

The way I got angry was exactly like my father… I knew where that anger was coming from. I just didn't want to work on it. Because I feel like if I worked on it, then I had to relive those memories. So it wasn't until I finally got the courage to go to therapy because I realized that I was becoming my father – and that's when my wife and I started talking about having a kid. (34b)

The major challenges that arose within couples due to maltreatment history were participants’ worries about stigma/judgment by their partner, negative emotions when comparing childhoods, and feeling inadequate as a partner or parent.

#### 3.2.1 Stigma/judgment

Some couples expressed a shared understanding of the stigma associated with experiencing maltreatment and the belief that this may make someone less suitable as a parent due to the repetition of certain patterns. Participant 36b expressed how being out in public with their crying baby was harder for her maltreated husband, as it triggered his fear of being perceived as a bad parent.

Others expressed worries about judgment coming specifically from romantic partners due to their maltreatment history. Participant 15b shared how his mother's infidelity was a difficult conversation to have with romantic partners and that partners viewed dating him as an “uphill battle” because of his issues with his mother. Participant 16b also shared receiving negative reactions when discussing his issues with his mother with a previous romantic partner. “She would very much ridicule me and criticize me. You know, like, ‘you must be misogynistic if you don't get along with your mom’ kind of stuff.’” Notably, none of the participants expressed feeling stigmatized or judged for their maltreatment history by their current partner.

Non‐maltreated partners shared that they were aware of concerns surrounding stigma. For instance, Participant 34b noted, “I think he thought if he came from this messed up family that, like, I wouldn't want to be with someone like that.” While some of these partners admitted that hearing about maltreatment history led to initial concerns that certain patterns may play out in how they communicated or handled conflict, these concerns were largely resolved by the time they had children.

#### 3.2.2 Comparing childhoods

Comparisons between each other's childhood experiences came up for seven couples. Participants described negative emotions, from jealousy to resentment to grief, arising from comparing childhoods. Participant 15b noted, “Comparing my parents to who they were, to who they should have been … just [leads to] being resentful, ruminating in it.” One participant shared negative feelings that come up when she is around her partner's family and how close they all are to one another—“I don't know whether [my negative emotions are because it] reminds me of something that I didn't like where I want more space or if it's that I regret that I don't have a family relationship like that” (10b).

#### 3.2.3 Feeling inadequate

In many couples, the participants with maltreatment history expressed feeling relationally inadequate, and these concerns became most salient when becoming a parent. However, one dyad demonstrated the asymmetry in how maltreated participants viewed themselves compared to how their partners viewed them as parents. Participant 15b shared “I am fearful—the fear is that I lack parental instincts, and she has them in spades”, while his partner believed “he does a great job [parenting], he just doesn't have the self‐confidence that he should because he does great with her” (15a). This dyad demonstrates the difficulty in convincing a partner with maltreatment history that they are doing a good job parenting. Feeling inadequate was powerful for many maltreated participants, and at times, their partners struggled to know how to help them overcome those beliefs. Although maltreated participants frequently expressed concerns about their own parenting adequacy, couples did not generally interpret non‐maltreated partners as inherently better equipped to parent. Instead, these concerns appeared to reflect internalized beliefs about competence by the maltreated partners that were negotiated within the couple. For example, while Partner 15b described fearing that he lacked parental instinct, Partner 15a attributed differences in the infant's attachment to situational factors (e.g., the infant's preference for the primary caregiver) rather than innate caregiving ability. Considered together, these accounts suggest that perceptions of inadequacy were not necessarily interpreted as reflecting true differences in parenting capacity, but rather as relationally negotiated beliefs that were recalibrated through coparenting experiences.

Expressing these feelings of inadequacy was, at times, difficult for participants. Several participants highlighted the barriers to seeking support and help from their partner or others, such as a therapist, including feelings of shame or avoidance.

There have been times where I know that I've got a response to certain situations that I take straight from [my caregivers]. And it's been hard for me to admit that and to communicate that to [my wife]…I'm ashamed to bring back up. (25b)

### 3.3 Theme 3: Dyadic support as co‐regulation for maltreatment‐related vulnerability

Couples described engaging in a range of strategies that functioned not only to provide practical support during the transition to parenthood, but to regulate vulnerabilities associated with childhood maltreatment within the couple relationship. Across dyads, these strategies appeared to facilitate emotional safety, reinforce parenting confidence, and support partners in enacting caregiving approaches that differed from those they had experienced in childhood. The word “stable” was used frequently in interviews when describing how their partner supports them, capturing the sense of emotional safety and structure that these partnerships provided, which contrasted the inconsistency and lack of safety that often existed in their childhoods.

[My wife is] able to, with regard to the wholeness of my experience and character and childhood, remain stable in her own roots. Whereas I think that's a difference from previous relationships that I've been in, as I trust my wife, implicitly and explicitly. And there was a lack of trust in those people to be ‐ I hate to quote this, but as Sheryl Crow said, strong enough to be my [partner], but that's the idea. (Participant 15b)

This sense of stability was not confined to participants with non‐maltreated partners—couples where both partners experienced maltreatment also felt a sense of stability in their partner. Further, while these interviews were focused on helping the partner with maltreatment feel supported and adjust to parenthood, most of these strategies helped the non‐maltreated partners and could be useful for all couples during this period.

#### 3.3.1 Partners as external regulators of self‐doubt

Across dyads, verbal affirmation and active listening functioned as mechanisms through which partners regulated maltreatment‐related self‐doubt during the transition to parenthood. Rather than serving as generic relational skills, these practices appeared to counteract deeply internalized beliefs about inadequacy that participants linked to their childhood experiences.

“When he's appreciative, or saying that he thinks that I'm a good mom, the kids love this, or this was so special – I told him that's the easiest way to make me feel supported and loved.” (16a)

Her partner's affirmation was not simply experienced as praise, but as corrective feedback that challenged doubts about her caregiving competence. Notably, participants frequently contrasted this affirmation with the absence of validation in their childhood homes, suggesting that verbal reassurance from partners carried particular emotional weight because it represented something previously lacking.

Listening similarly functioned as a regulatory practice within dyads. Participants described their partners’ willingness to witness their histories without attempting to fix or minimize them as profoundly stabilizing. As Participant 16a explained: “He will be a good listener… there's nothing you can do to like, fix it, or undo it.”

The emphasis on witnessing rather than solving appeared to be especially meaningful in the context of maltreatment, where participants often described feeling unheard or dismissed in childhood. In this way, listening operated not only as supportive communication but as a relational experience that validated participants’ histories and reduced isolation during the transition to parenthood.

Together, these dyadic processes suggest that affirmation and listening functioned as forms of co‐regulation, helping maltreated participants recalibrate internalized beliefs about parenting adequacy as they entered new caregiving roles.

#### 3.3.2 Accountability as a preventative parenting strategy

Another helpful strategy utilized by couples was holding each other accountable in meeting parenting expectations for themselves and their families. Participants with maltreatment shared that they felt more confident in their ability to parent differently than they were parented because they trusted their partner to call out any problematic behavior:

I've got a really good partner that helps hold me accountable. And I feel like I do the same for her. So, it's not daunting at all to me to know that I've got somebody that is never afraid to say exactly what's on her mind. (25b)

Participant 34b similarly stated, “One of the things she said once was ‘The only way you can be your father is if you don't work on yourself.’ And I think that's one of the things that really stuck with me… So, I am going to work on myself.” Several participants described nuance to making this strategy feel supportive rather than eliciting defensiveness. For instance, Participant 16b noted, “I don't ever do that, ‘you're acting like your mom’ thing, but I'll be like, ‘you're not anxious because of anything I've said or because of anything you're doing that's wrong. Where's that coming from?’”

In dyads in which both partners reported childhood maltreatment histories, support was less frequently described as one partner stabilizing the other and more often as a shared commitment to monitoring relational patterns that may mimic those experienced in childhood. In these couples, accountability practices were described as reciprocal rather than corrective, suggesting that co‐regulation was enacted through mutual attention toward avoiding intergenerational repetition.

#### 3.3.3 Joint boundary formation and holding

The final strategy that was discussed by most couples was the co‐creation of boundaries and supporting each other in holding those boundaries. Many maltreated participants were still in contact with caregivers, and having a child often meant renegotiating what that relationship would look like. Many participants described finding it necessary to put boundaries in place between their caregivers and themselves/their child, but this could be difficult as this process could trigger memories of feeling helpless or unsafe as a child around their caregivers. A major way that partners showed support was by helping to set and hold these boundaries, whether through discussion of what boundaries would look like, helping their partner process their emotions in navigating their caregiver's and child's interactions, or enforcing the boundaries with their partner's caregivers.

Several non‐maltreated partners described their experience of helping to hold these boundaries and being willing to step in and help their partner negotiate what that boundary should look like if necessary. Participant 25a described this process:

[My partner's parents] have very little to no contact with our baby because he doesn't want that toxicity like bleeding into him. And I've always said whatever relationship he wants with them is the relationship that he should and can have. But if he feels it's best for like our child to not be involved with them, then that's his call. If there comes a point where I feel like I need to step in or if I feel like he's making the wrong call, I guess then I would put my foot down.

Among couples in which both partners had experienced maltreatment, participants sometimes described how their own childhood experiences increased their sensitivity to their partner's relational distress and strengthened their commitment to supporting boundary formation and maintenance. In some cases, witnessing the long‐term emotional impact of abusive family dynamics in their own families appeared to motivate participants to actively support their partner in establishing boundaries. As one participant reflected, “It honestly, I think it gave me the foresight to give my wife full support…Seeing how [abuse] affected my mom, I didn't want my wife to suffer or deal with those emotional heartaches and hardships the way my mom did” (61b).

### 3.4 Theme 4: Couples’ resilient and intentional orientation towards family life

The final theme reflected couples’ description of jointly reorienting their lives around family in ways that reflected shared efforts to construct a parenting approach distinct from their childhood experiences.

I've never been more tired, but also never been happier and had more love and like felt more like satisfaction – simply everything's got to grow. I've never felt more in love with my life. Just the range of experience has gotten bigger. (49b)

#### 3.4.1 Sense of belonging as coparent

Across dyads, partners described developing an increased sense of emotional belonging within their relationship following the birth of their child, often framed as a shared responsibility in navigating early parenthood. “If anything, it's actually probably made us closer in the sense that even though we don't talk about it openly, we just definitely feel like we are the team, like it's me and her against the world” (16b).

This participant elaborated how this feeling of unity after becoming parents may have related in part to their partner's maltreatment history because it was perhaps the first time, she felt a sense of belonging in a family system or thought of family as a supportive entity. This “us vs. the world” phenomenon seems heightened for these couples when contrasted to the maltreated partner's experience growing up or to their current relationship with those caregivers.

This new connected feeling was also related to an acute awareness of how much support they need from their partner in raising a child.

I feel closer [to my partner], like we are bound through this experience. We need each other to raise our child… Recognizing how important having the other part of the partnership is for my ability to navigate this challenge has been very, very evident to me. And so recognizing how much I need and depend on having a partner has been a lot more prominent in my life. (10b)

Together, these accounts suggest that dependence on one another in navigating early caregiving demands contributed to a shared sense of partnership that was experienced as protective during the transition to parenthood

#### 3.4.2 Jointly rewriting family narrative

Across several couples, partners described jointly reflecting on whether and how aspects of their childhood caregiving environments would inform their own parenting practices. Some described a growing confidence in themselves after finding their fears about repeating old patterns did not come to fruition.

That anxiety and scary feeling of ‘What if I do become my dad?’ kind of just like vanished. … I don't ever see myself treating her the way my parents did. It's so obvious to me that like, you're not your father anymore, you'll never be. (34b)

Couples described collaborative decision‐making regarding parenting strategies that worked for them as a family from various sources and models. Friends with children, faith‐based leaders and teachings, their own extended families, and simply doing the opposite of things their caregivers did, were all models mentioned. Couples did not generally interpret childhood maltreatment history as suggestive of poorer parenting capability. Instead, parenting was described as a collaborative process through which both partners could develop new caregiving practices regardless of prior experience. Participant 15b explained this phenomenon well with an analogy.

My main instinct is like to go to the grocery store with what we can learn about our life. Like, ‘Oh, that looks great. I want to try that’. And if you try it and it works for you, then I think you continue to cook with that ingredient. And if it doesn't, then you don't, but it isn't like trade in my parents for her parents.

This belief allowed maltreated participants to feel skilled and valued in coparenting and helped to reduce the stigma around being ill‐equipped to parent due to a maltreatment history.

#### 3.4.3 Moving forward with intention

Many couples described co‐parenting with intentionality. Approaching parenting with this level of planning and structure was perhaps more common due to the anxiety about repeating previous maltreatment patterns.

One thing that I love about [Partner] and the way that he chooses to go about parenting is that he actively chooses – like when I was pregnant, he had several very vulnerable and emotional moments where he was like, ‘I'm like my parents. I'm scared if that's all I know, that's all I can be’ type of thing. And so, I love that he's thinking about that type of thing, because it's obvious that that's not what he wants. Like he wants to be better, and he wants to be different for our kids. And he obviously exceeds all expectations. (25a)

Other participants with maltreatment histories explained the preparation and effort that they put in to make sure that they did not continue the cycle of maltreatment. Often, this involved handling emotions or responding during conflict differently. For instance, Participant 23b stated, “I'm so desperate to not make the same mistakes that my parents made, that it's easy for me [to calm down during conflict], like, especially with the baby in the picture too.” Partners frequently described encouraging or reinforcing these efforts, framing intentional parenting as a shared commitment within the couple.

Across dyads, resilience in the context of maltreatment was often described as emerging through shared commitment to enact different caregiving practices within their own family systems.

All the [messed] up [stuff] that I went through as a kid, you know, I think that if I didn't go through it, I wouldn't be the parent that I am today. I think that all the [things] that I went through made me a better husband and made me a better father. (34b)

## 4 DISCUSSION

The present study examined how couples navigated the transition to parenthood in the context of childhood maltreatment history, with particular attention to how partners jointly made sense of earlier caregiving experiences and supported one another in assuming new caregiving roles. Findings suggest that the influence of childhood maltreatment on adjustment to parenthood may be best understood as a relational process, negotiated within the couple as partners prepare for and enact caregiving responsibilities. Across dyads, partners contributed to identifying, validating, and reinterpreting earlier experiences of maltreatment, and these shared meaning‐making processes appeared to shape how couples anticipated and responded to parenting‐related challenges.

Consistent with Theme 1 (Relational Meaning‐Making), participants described partners playing an active role in helping to name or reinterpret earlier caregiving experiences that had previously been minimized or normalized. In some dyads, this process involved gentle inquiry (e.g., asking where certain emotional responses originated), whereas in others, partners more directly drew connections between present‐day parenting behaviors and participants’ experiences with their own caregivers. Considered together, these accounts suggest that partners functioned as relational mirrors through which earlier caregiving models could be identified and reconsidered. In several cases, this appeared to contribute to a sense of stability within the relationship, described by participants as emotional consistency, predictability in responses during conflict, or a shared ability to pause and reflect on whether a reaction reflected current parenting values or earlier experiences.

In line with past literature (e.g., Belsky & Isabella, 1985; Chamberlain et al., 2019a), couples with childhood maltreatment histories experienced unique challenges in their relationships or transitioning to parenthood. Participants with maltreatment histories frequently expressed fears about repeating negative caregiving patterns; however, partners did not generally interpret these concerns as reflecting inherent differences in parenting ability. Instead, concerns about adequacy appeared to reflect internalized beliefs about competence that were negotiated within the relationship. In several dyads, partners attributed perceived differences in caregiving confidence to situational factors (e.g., infant attachment patterns or caregiving experience) rather than to stable differences in parenting ability, suggesting that parenting competence was constructed over time rather than inherited.

Theme 3 highlighted the ways in which partners helped to co‐regulate emotions and behaviors that arose during the transition to parenthood. Partners described engaging in a range of supportive behaviors that appeared to regulate vulnerabilities associated with anticipated caregiving roles. Verbal affirmation, active listening, and accountability were experienced not only as supportive communication practices, but as mechanisms through which partners helped one another manage parenting‐related self‐doubt linked to earlier caregiving experiences. Similarly, jointly setting and maintaining boundaries with families of origin was described as a relational process, with partners assisting one another in navigating emotionally complex decisions regarding ongoing contact with caregivers who had previously been sources of harm. This regulatory dynamic sometimes differed in dyads where both partners reported maltreatment histories, where support was described less as one partner stabilizing the other and more as a shared effort to monitor relational patterns that might mirror those experienced in childhood. These findings are consistent with attachment‐based perspectives suggesting that supportive adult relationships may facilitate revision of earlier working models in the context of new caregiving roles (Rholes et al., 2021; Simpson et al., 2003).

Broadly, dyads tended to report a resilient and intentional outlook towards parenting and family life (theme 4). Attachment theory suggests early caregiver relationships shape attachment styles into adulthood (Fraley & Roisman, 2019). While maltreated individuals often develop insecure attachment styles, life experiences that contradict early working models, such as a secure romantic relationship, can foster more secure attachment (Rholes et al., 2021; Simpson et al., 2003). This may explain participants’ largely positive outlooks. Despite having experienced maltreatment, many saw the perinatal period as an opportunity to collaboratively rewrite their narrative and do things differently than their caregivers had. Many participants viewed their relationships as the stable base from which they could create this new narrative, and very few held deep concerns about repeating old patterns once their child was born. Although many had anxiety earlier in their lives about relationships or parenting due to their upbringing, being able to build a successful partnership and navigate the challenges of the perinatal period together instilled confidence that they could break the cycle of maltreatment.

### 4.1 Limitations and future directions

Several limitations should be considered when interpreting these findings. The present study focused on couples in long‐term, committed relationships in which both partners were willing to participate in a joint research study. Given that childhood maltreatment has been associated with later difficulties in relational functioning (Zamir, 2022), it is possible that individuals most significantly impacted by their maltreatment histories were less likely to be represented in this sample. Participation also required both partners to consent to involvement, which may have further limited the inclusion of couples experiencing higher levels of relational distress or instability. Severe childhood maltreatment is often associated with broader functional difficulties later in life (Kim & Cicchetti, 2010; McLaughlin et al., 2020), not solely relational challenges, and thus the present sample may not reflect the full range of adjustment difficulties experienced by parents with maltreatment histories. While this limits generalizability, the study aimed to examine dyadic processes through which couples navigate the transition to parenthood in the context of childhood maltreatment within ongoing relationships.

Because participation required both partners to be aware of and willing to discuss childhood experiences within the context of a joint interview study, these findings reflect the experiences of couples in which maltreatment history had already been disclosed within the relationship. Future research may benefit from exploring the experiences of parents who have not disclosed maltreatment histories to their partners through individually focused methodologies that do not involve partner participation, given the ethical and relational complexities inherent in studying non‐disclosure within dyadic designs.

Finally, restricting participation to English‐speaking couples limited our ability to examine how cultural or linguistic factors may shape the meaning‐making of childhood maltreatment and its influence on coparenting adjustment. Future research would benefit from explicitly examining the role of culture in how maltreatment is interpreted across generations and within couple relationships during the transition to parenthood.

### 4.2 Implications for clinical practice

This study expands our understanding of how maltreatment shapes the transition to parenthood within romantic and coparenting relationships. While previous research emphasizes risks associated with maltreatment, our findings highlight how relational processes can support adjustment to parenthood.

Early disclosure of maltreatment and partner validation helped alleviate fears about being unfit as parents. In clinical settings, therapists may consider supporting couples in discussing how past experiences shape expectations for coparenting to encourage open dialogue about maltreatment histories. Participants also often found discussing their histories in interviews to be cathartic, suggesting that safe spaces for reflection and meaning‐making are clinically valuable.

Participants described several relational practices that helped them navigate insecurities related to parenting after childhood maltreatment. In particular, partners frequently served as external regulators of self‐doubt by offering reassurance about parenting competence and validating concerns that stemmed from earlier caregiving experiences. Many participants described partners’ listening as especially meaningful because it created a space in which their maltreatment histories could be witnessed without needing to be “fixed.” In clinical settings, therapists may consider supporting couples in developing similar practices, such as helping partners recognize moments when reassurance, attentive listening, or emotional validation may be more helpful than problem‐solving when addressing parenting‐related anxieties.

A novel finding was how instrumental partners were in aiding individuals with maltreatment histories in interpersonal effectiveness with their families of origin. Interventions for couples with maltreatment histories during this transition may benefit from including discussions about what boundaries with caregivers may look like and a general focus on interpersonal effectiveness skills, such as those taught in dialectical behavior therapy (DBT; Linehan, 2015) to help both partners feel equipped with the tools they need to enforce the boundaries they feel necessary for their new child.

Although prior research on childhood maltreatment and the transition to parenthood has focused primarily on mothers, our inclusion of mother‐father dyads allowed us to examine fathers’ experiences as well. Findings suggest that relational processes of support and meaning‐making may serve protective functions for both mothers and fathers with maltreatment histories, extending prior work on the transition to motherhood to the transition to fatherhood as well (Chamberlain et al., 2019a; Christie et al., 2017).

This interpretative phenomenological study explored how couples with childhood maltreatment histories navigate the transition to parenthood. Four interrelated dyadic processes were identified: relational meaning‐making of childhood maltreatment, relational challenges activated by maltreatment history, dyadic support as co‐regulation for maltreatment‐related vulnerability, and couples’ resilient and intentional orientation towards family life. Together, these findings highlight how experiences of childhood maltreatment are not negotiated in isolation during the transition to parenthood, but are jointly interpreted and managed within the couple relationship. Through shared reflection, collaborative boundary setting, and mutual support in navigating parenting‐related concerns, couples described working together to construct caregiving approaches that were distinct from their own childhood experiences and set a new course for their family.

## CONFLICT OF INTEREST STATEMENT

The authors declare no conflicts of interest.

## FUNDING INFORMATION

The authors report no funding.

## Supporting information




Supporting Information

## Data Availability

Data supporting the findings of this study are available from the first author upon reasonable request.
