# Supplementary material for: “Desperate not to make the same mistakes”: Couple adjustment to parenthood in the context of childhood maltreatment
Source: Infant Ment Health J. 2026 Jun 15;47(4):e70106. doi: 10.1002/imhj.70106 (PMC13269663; doi:10.1002/imhj.70106)
Supplement: Supplementary file 1 — Supporting Information [file IMHJ-47-0-s001.docx]

**Appendix A.** Interview Guide

1. Describe your upbringing/relationship with parents or caregivers.
   1. Do you talk about your upbringing with your partner often?
   2. How have conversations around your upbringing with your partner gone?
2. Have you been thinking more about your childhood/upbringing during this time? What is that like?
3. Do you think your upbringing informs the way you parent your child? How?
4. Describe your partner’s upbringing/relationship with parents or caregivers.
   1. Do they talk about their upbringing often?
5. Do you think that their upbringing informs how they parent your child? How?
6. How has your relationship been during this period? What changes have you noticed in how you two interact?
   1. Do you feel supported by your partner in the relationship? As a parent?
7. Do you have discussions about how you parent the child/what type of parents you want to be? How do those discussions usually go?
8. How happy are you in your relationship?
9. How happy are you with the way you are coparenting your child?
   1. How do you feel about the division of labor between you and your partner when it comes to caring for your child?
10. Any other things you want me to know?

**Appendix B.** *Representative Quotes*

|  |  |  |
| --- | --- | --- |
| **Relational Meaning-Making of Maltreatment** | | |
| Minimizing maltreatment | There wasn't any sort of physical abuse or physical neglect or anything like that that ever occurred at home. Like I never felt I was left alone for extended periods of time or there was never any sexual abuse. I've never had any sort of molestation or anything like that. Basically, the only thing that kind of bugs me a little bit and it's kind of impacting me with my parenting is my dad. He was born in the late 40s, like at the very end of the baby boomer spectrum type thing. And he was very much a disciplinarian. So, like, if, you didn't do things right or if things didn't go a certain way, like we had - I'm not going to say, like physical but the belt was an option that was often used as a kind of a mental threat. | 36a |
| Minimizing maltreatment | There was never any like, “I'm gonna beat you to death” abuse. But she would like … unnecessarily spank us to the point where I literally do vividly remember that. | 61a |
| Partner-led identification of maltreatment | I think that people think that whatever they were brought up with is normal, right? That's something like whatever they're familiar with is normal. So, like, I would just say, like, oh, like, obviously, like, my mom's doing this because this is normal. And then he'd be like, ‘oh, like, actually, I used to do it this way’ or ‘my mom used to do it this way’. And I'm like, Yeah, [my experience was] not normal. | 49a |
| Emotional reaction to partner's history | It has made me more appreciative, I guess, feel more lucky. You know, I feel like I'm in the minority of people that don't really have much regret or, at least, angst about my relationship with my parents, not that my childhood was perfect…I have insecurities and all those sorts of things, but I don't trace any of those to my parents. So, you know, that's sort of reflecting on myself, I feel lucky and I feel sad and empathetic towards her for her experience. | 10a |
| Emotional reaction to partner's history | I think the one thing that sticks out is he has this memory where he made it to the state finals with tennis and no one in his family showed up to watch him and that's really hard for me to hear where it's like, I feel so incredibly sad for him, and I feel angry at his parents. It helps me understand more of like why he is at every single one of our son’s soccer games. So, there's sort of a feeling of being protective of him and being sad that someone I love had to be hurt like that. | 16a |
| **Relational Challenges Activated by Maltreatment History** | | |
| Stigma/Judgment | I think because he doesn't want to be judged as a parent. Especially when our daughter was like really little like he would feel like if we went out in public like well, ‘I don't want people to think this’ and I'm like, ‘well she's gonna cry’. But I think that part of that like feeling judged piece comes up for him more now that we have a kid and then hearing [his mom] say things like, just about like how we parent. It I think it does hit some harder. | 36b |
| Comparing Childhoods | [My partner] has a really good relationship with her family. And I think that was one of the things too, like seeing the way – I think my childhood is really, I'm sorry using this word, [messed] up. Kind of putting her in mind, side by side, it kind of looks like she had a really good childhood. So, I feel like when I first met her I was like, my god, you had the best childhood, you know, like, your parents were always there. I feel like she'll tell me stuff like block parties and like their parents used to like make them costumes and all that stuff… I think that [comparing childhoods] was really bad for me to do. Like when she'll share some things, I'll be like, “Oh, but you were never hit by a belt. So you're fine.” You know like, “What do you mean your mom was always home when you got home from school?”…I think that's when she got upset. She's like, “no, even though yours was worse, mine was still valid.” | 34b |
| Feeling Inadequate | I remember thinking, ‘why do you text me every day?’ Like I started like, pushing him off a little bit. I was like, why does he care? And I realized that I did that because I had that like unconscious feeling that I just wasn't worth it. You know, because my mom was never like, ‘how do you feel? What do you want to do today?’ She never asked, she just did whatever she wanted. But then here comes my husband being like, ‘Hey, how do you feel?’…You know, like, actually dare I say, bothering to care? So that was new to me. | 61a |
| Feeling Inadequate | But here I am as a dad, and that's like the one place that seems to be kicking my ass. And my wife insists that she can't help me see that I'm a great dad and that I have to see that for myself. She can't make me see that I am a great partner to her. I have to see that for myself. And yet, there's this part of my brain thinking, “you could be doing more”. | 15b |
| **Dyadic Support as Co-Regulation** | | |
| Partners as external regulators of self-doubt | I never really got positive feedback from [my mom] saying ‘You're doing so good. I'm so proud of you.’ My husband was the one that was like ‘you're doing really well.’ Even my in-laws were like, ‘We're proud of you.’ You know, it's that positive verbal affirmation that I want to instill in my child and future children. | 61a |
| Accountability as preventative parenting strategy | I definitely think that we feel closer as just a unit in terms of trusting each other with a baby and trusting each other's if one of us is like, “no, I feel very strongly we should do this this way” because she's not feeling well or we need to call a doctor or whatever, we've been pretty good about trusting the other person and listening. | 15a |
| Joint boundary formation and holding | There was just a lot of stuff that happened where I would – I got knocked around a couple of different times. Or I got beaten down verbally [by my caregivers], like told how I'm not gonna be able to get anywhere in life and that was really hard for me. And so, after I met my wife, she encouraged me to leave that situation. | 25b |
| Joint boundary formation and holding | We [have conversations about the level of involvement that we want his parents to have in our child’s life]. I feel like, it's his parents, so it's his choice. Then like with my parents, it's my parents, so I feel like it's a little bit more my choice in that regard. So I kind of like try and go off of what he wants and just support his choice. | 34a |
| **Couples' Resilient and Intentional Orientation Toward Family Life** | | |
| Sense of belonging as coparents | Some weeks are actually much better, like, it's like a team. There's like more of a team aspect where we're way more united, so the uniting makes us feel closer. | 23a |
| Sense of belonging as coparents | I think we felt closer in a lot of ways. You know, especially like going through the NICU experience, as kind of the opener to beginning the parenting journey because it was kind of a like, you know, a big emotional challenge that we kind of faced together. And we're like both going through the exact same thing together in a way that like no one else in our lives was. I think I've like I've gained new respect and like appreciation for [Partner]. | 42b |
| Jointly rewriting family narratives | I think we both acknowledge the strengths and weaknesses of our parents and how we were raised. I think we're both trying to be what we want to be as a parent versus what our parents were. Like, what are the strengths and weaknesses and what do we as a couple feel passionately as parents. Like, what is most important to us that she remembers. | 15a |
| Jointly rewriting family narratives | I think it just took time, in our years of marriage and before we were married, to kind of go through the cafeteria of our upbringing and figure out what we wanted to keep and what we wanted to leave. And it's a mixture. There are there are things that my wife's culture and ethnicity that added to my life that I never would have thought of, or never would have experienced, but enrich life and make us more successful people. | 38a |
| Jointly rewriting family narratives | We just had to come back to that, like my family didn't have as many like specific traditions just because my grandparents like when they came to America, they weren't observant and her grandparents were the observant ones and they were traditional, so like finding the right traditions and customs. The more minor part of our day-to-day life was like, wasn't like a conflict, but it was something that we had to work out and kind of figure out and it was something that we've learned to kind of blend. We kind of tried to take the beauty from both and particularly like from our own personal lines we kind of make our own little blend. | 49b |
| Moving forward with intention | I think a lot about how I will handle conflict, how will I handle big emotions, both mine and my kids, and I think I have intentionality about that. I don't know what the execution will end up looking like. I think having thought about it in advance and try to figure out what the tools are and like read up a little bit on like, how do you deal with a kid having a tantrum... I think because of the way I was parented and wanting to have a more well-rounded toolkit to bring to those challenges than I think that my parents did. | 10b |
| Moving forward with intention | I ask myself the question that is, “is that the energy that I would want to have in our household?” and the answer is absolutely not like that. That’s exactly the reason that I've pursued counselling in this regard. There are things that I observed my dad do with women that I made a conscious choice, I'm never going to do that. And so, I don't, because I've kind of built up this ability to do something else. | 15b |
